# Association between early heart rate trajectories in post-PCI STEMI patients and prognosis after hospital discharge

**DOI:** 10.1080/07853890.2025.2468267

**Published:** 2025-02-22

**Authors:** Dan Wu, Yanping Yin, Jing Zheng, Xiaoshan Zhou, Fanli Cheng, Yiran Wang, Kaini Li, Xuanting Mou, Wenting Lin, Chao Feng, Sixiang Jia, Weili Ge, Shudong Xia

**Affiliations:** ^a^ Department of Cardiology, The Fourth Affiliated Hospital of School of Medicine, and International School of Medicine, International Institutes of Medicine, Zhejiang University, Yiwu, China; ^b^ Department of Cardiology, Taizhou Hospital of Zhejiang Province Affiliated to Wenzhou Medical University, Linhai, China; ^c^ Laboratory of Cardiovascular Disease, Taizhou Hospital of Zhejiang Province Affiliated to Wenzhou Medical University, Linhai, China; ^d^ The Quzhou Affiliated Hospital of Wenzhou Medical University, Quzhou People’s Hospital, Quzhou, China

**Keywords:** Heart rate trajectory, major adverse cardiovascular events, ST-segment elevation myocardial infarction, ventricular remodelling

## Abstract

**Background:**

Timely percutaneous coronary intervention (PCI) is crucial for restoring myocardial blood supply in ST-segment elevation myocardial infarction (STEMI) patients, with the first 72 h being a critical period for early ventricular remodelling. The association between heart rate trajectories within this period and after hospital discharge outcomes in STEMI patients post-PCI remains unclear.

**Methods:**

We conducted a retrospective study involving STEMI patients who underwent successful PCI at three tertiary hospitals in Zhejiang Province, China. Heart rate data were collected every 8 h post-PCI through nursing records, along with intraoperative findings and biochemical markers. Using trajectory modelling, we identified heart rate patterns at 24, 48 and 72 h post-PCI, determined the optimal number of trajectory groups using Akaike information criterion (AIC) and Bayesian information criterion (BIC) criteria, and performed a minimum 3-month follow-up. Cox regression analysed the association between early heart rate trajectories and major adverse cardiovascular events (MACEs) post-discharge. The prognostic value of trajectory models was assessed using the area under the curve (AUC).

**Results:**

A total of 1257 patients were included, with an average follow-up duration of 28.72 ± 21.14 months and a mean age of 60.42 ± 14.19 years; 1013 (80.59%) were male. Growth mixture modelling identified four distinct heart rate trajectory groups at 24, 48 and 72 h post-PCI. Higher heart rate trajectories with rates greater than 80 bpm were strongly associated with MACEs, and the 72-hour heart rate trajectory showed a predictive value for MACEs (AUC = 0.745, 95% CI: 0.709–0.781).

**Conclusions:**

Elevated heart rate trajectories exceeding 80 bpm within 72 h after PCI are associated with an increased risk of MACEs post-discharge. Heart rate management should be further emphasized in post-PCI STEMI patients.

## 1. Introduction

Coronary artery disease (CAD) remains a leading global health burden, with its incidence and mortality rates continuing to rise [1–3]. ST-segment elevation myocardial infarction (STEMI), as the most severe and life-threatening acute phase of CAD, necessitates urgent reperfusion therapy, which offers a critical opportunity to salvage myocardial tissue [1,4]. Effective percutaneous coronary intervention (PCI) can significantly minimize infarct size and improve survival outcomes post-discharge in STEMI patients [1,5].

The prognosis of STEMI patients is contingent not only upon timely and effective reperfusion therapy but also on the management of risk factors. Patients who have sustained an acute myocardial infarction (AMI) inevitably undergo ventricular remodelling, and suboptimal risk factor management can accelerate this process [6]. The role of post-PCI heart rate control in determining patient outcomes has been increasingly recognized [7,8]. Heart rate, a fundamental physiological parameter readily obtainable in clinical practice, reflects the balance between the sympathetic and parasympathetic nervous systems and is significantly related to myocardial oxygen consumption and coronary blood supply [9,10]. Strong evidence links heart rate with prognosis in AMI, highlighting its potential for risk stratification in mortality [11,12]. Post-PCI heart rate may be affected by factors such as myocardial injury, coronary lesions and pharmacological treatments. Despite this, most research has focused on single-point heart rate measurements, overlooking the dynamic nature of heart rate fluctuations. Although heart rate variability (HRV), as assessed by 24-hour Holter monitoring, offers insights into variations in successive heartbeats and has been associated with mortality in early studies, HRV does not fully capture the direction and magnitude of heart rate changes [13–15].

Trajectory modelling is an analytical approach that utilizes multiple repeated measures to group individuals based on the dynamic changes in a specific parameter, thus addressing the limitations of single-point data analysis [16,17]. Applying trajectory modelling to the study of heart rate dynamics may provide novel clinical insights for managing STEMI patients post-PCI. Wei et al. have demonstrated the predictive value of heart rate trajectory groups in heart failure populations, while Wang et al. have identified correlations between 72-hour heart rate trajectory patterns and outcomes in cardiogenic stroke [18,19]. The initial 72 h post-myocardial infarction are recognized as an early phase of myocardial remodelling following myocardial infarction, yet the association between heart rate trajectories during this period and prognosis in STEMI patients remains unclear [20]. Thus, specific patterns in early heart rate trajectories following PCI may indicate varying prognostic outcomes. This study seeks to investigate the relationship between early heart rate trajectory changes after PCI and the incidence of major adverse cardiovascular events (MACEs) post-discharge in STEMI patients.

## 2. Methods

### 2.1. Study patients and definitions related to the study

This study is a retrospective cohort analysis. Participants were recruited from three tertiary referral centres in Zhejiang Province: The Fourth Affiliated Hospital of Zhejiang University School of Medicine (2015.11-2024.3, *n* = 725), Taizhou Hospital of Zhejiang Province (2019.1-2023.12, *n* = 672) and Quzhou People’s Hospital (2022.1-2023.1, *n* = 340). The cohort comprised patients with a diagnosis of acute STEMI who had undergone successful coronary artery intervention. All interventions were performed by experienced interventional cardiology teams at each institution, adhering to established guidelines for myocardial revascularization.

This research has received approval from the leading Ethics Committee of The Fourth Affiliated Hospital of Zhejiang University School of Medicine (Approval Number: K2024149). The study adheres to the Declaration of Helsinki. Due to the retrospective design of the study, the ethics committee waived the requirement for informed consent from eligible patients.

#### 2.1.1. STEMI

Refers to myocardial cell necrosis caused by myocardial ischemia, clinically characterized by an elevation of myocardial injury biomarkers (such as cTnT, cardiac troponin T) above the 99th percentile of the upper reference limit, along with evidence of myocardial ischemia, including new ischemic changes on the electrocardiogram (such as significant ST-T changes or left bundle branch block).

#### 2.1.2. Endpoint events

Major adverse cardiovascular events after discharge include cardiogenic death, non-fatal myocardial infarction, recurrent angina, unplanned revascularization, recurrent heart failure, arrhythmias and stroke.

#### 2.1.3. Inclusion criteria

The study included patients diagnosed with acute STEMI attributable to vulnerable plaques (rupture or erosion), as confirmed by coronary angiography, in accordance with the Fourth Universal Definition of Myocardial Infarction (2018) [21]. The demographic and clinical data of these patients were extracted from the electronic medical record systems of the aforementioned three hospitals.

#### 2.1.4. Exclusion criteria

- Patients with implanted cardiac pacemakers or other anti-arrhythmic devices.
- Patients with STEMI who declined emergency PCI during hospitalization.
- Patients who underwent primary culprit vessel revascularization via PCI with ECMO support for life maintenance during hospitalization; patients who experienced MACEs during hospitalization following primary culprit vessel PCI; patients who did not attend regular outpatient follow-up or were unwilling to participate in telephone follow-up inquiries after PCI.
- Patients with severe comorbid conditions, such as refractory hypoxemia, severe hepatic or renal dysfunction, severe infections, or end-stage malignancies.

**Figure 1.**
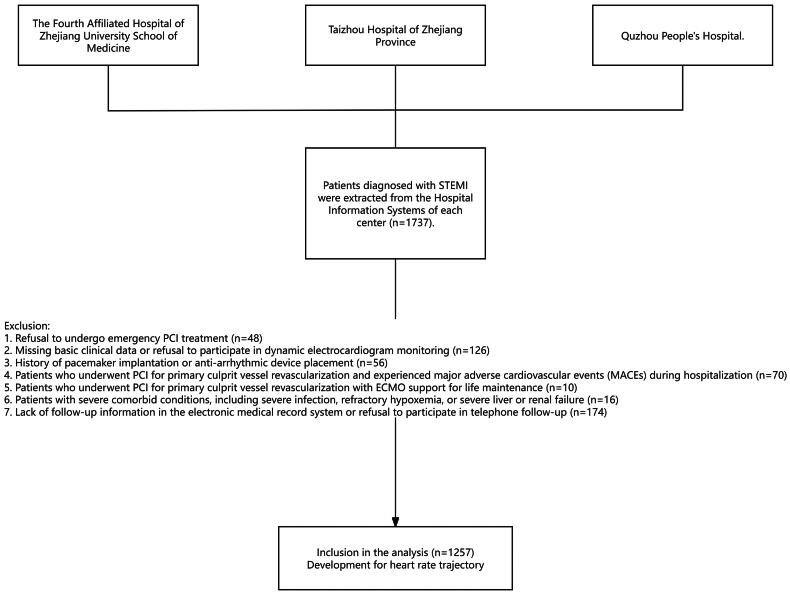
Flowchart of this study.

Following the application of these criteria, the final study cohort comprised 1257 patients. Figure 1 is the flowchart of this study. For patients meeting the study criteria, we conducted follow-up for no less than 3 months, with the follow-up period extending through June 2024.

### 2.2. Data collection and follow up

The study primarily recorded the following data: demographic information of the study population, heart rate recordings within 72 h post-PCI, cardiovascular risk factors, medical history and medication information, coronary angiography (if the degree of coronary artery stenosis is equal or greater than 50%, it is considered to be significant stenosis) and PCI procedure indices, and laboratory test results. Demographic data included basic parameters such as age, sex, height, weight and body mass index. Heart rate within 72 h post-PCI refers to values recorded by electrocardiographic monitoring or by nursing staff following coronary artery intervention. Cardiovascular risk factors encompassed history of smoking and alcohol use, diabetes and hypertension. Medical history and medication information (post-revascularization completion) included antiplatelet therapy, calcium channel blockers (CCBs), angiotensin-converting enzyme inhibitors (ACEIs) or angiotensin receptor blockers (ARBs), β-blockers and statins. Laboratory tests involved collecting blood samples the morning after admission, with an eight-hour fasting period generally required. Standardized laboratory techniques were used to measure biochemical parameters, including serum levels of lipids, liver function markers, renal function markers, cardiac enzyme profiles and glucose.

#### 2.2.1. Follow up

For patients who have experienced acute coronary events, due to the heightened risk during the ‘vulnerable period’ within the first year post-discharge, we recommend follow-up at least once every 1–3 months, as outlined in the discharge report. For patients who remain stable after one year, follow-up intervals are extended to every 6–12 months. Follow-up records are accessible via the electronic medical records system, enabling efficient data quality control for our study. In the absence of follow-up information in the system or during the follow-up period, the patient’s current survival status is confirmed through telephone follow-up.

### 2.3. Statistical analysis

The analysis was stratified based on the occurrence of MACEs post-discharge in STEMI patients. Descriptive statistics for normally distributed continuous variables were expressed as mean ± SD, and comparisons were made using the independent two-sample *t*-test. For non-normally distributed continuous variables, data were expressed as *M* (*Q*1, *Q*3) and analysed using the Mann–Whitney *U*-test. Categorical data were presented as *n* (%), with differences assessed using the Chi-square test.

Group-based trajectory modelling (GBTM) was conducted using the ‘traj’ command in Stata 18.0 (StataCorp, College Station, TX) to determine the heart rate trajectories of STEMI patients at 24, 48 and 72 h post-PCI. We fitted models with 1–5 trajectories for each time point and evaluated the significance of intercept, linear, quadratic and cubic terms. The optimal model was selected based on the Bayesian information criterion (BIC), Akaike information criterion (AIC), entropy and average posterior probability (AvePP). Specifically, the optimal model was determined by: (1) minimizing BIC and AIC for better fit; (2) achieving entropy ≥0.7, with values closer to 1 indicating better fit; (3) AvePP >0.7 indicating acceptable model fit; and (4) ensuring a minimum sample size of 2% per trajectory group. After determining the optimal number of trajectories, polynomial degrees were gradually reduced until all group polynomial terms had *p* values <.05.

Kaplan–Meier’s survival curves were used to assess the incidence of MACEs across the 24 h, 48 h and 72 h heart rate trajectories in STEMI patients, with group comparisons performed using the Log-rank test.

Multivariate Cox proportional hazards models were constructed to evaluate the impact of average heart rate at 24 h, and heart rate trajectories at 24 h, 48 h and 72 h on MACEs. Three models were developed: model 1 (unadjusted), model 2 (adjusted for age, gender and BMI) and model 3 (after adjusting for variables such as age, gender, BMI, SBP, DBP, smoking, drinking, hypertension, COPD, atrial fibrillation, tumour, myocardiopathy, diabetes, stroke, old myocardial infarction, Killip class, NT-proBNP, LDL, triglyceride (TG), creatinine, expired myocardial infarction, LVEF, left main artery (LM), left anterior descending coronary artery (LAD), LCX, right coronary artery (RA), other branches, inpatient days, cTnT, CK-MB, βBlock, CCB and ACEI/ARB).

The effects of average heart rate at 24 h, and heart rate trajectories at 24 h, 48 h and 72 h on MACE were explored across subgroups defined by age, gender, Killip class, history of myocardial infarction, RA, β-blocker use and inpatient days, with interaction *p* values calculated. We plotted ROC curves based on the maximum survival time of the population with MACEs in this study. The predictive performance of different heart rate trajectories was evaluated by comparing the area under the curve (AUC) using the DeLong test. The DeLong test was applied to assess whether the differences in AUCs between the ROC curves of the 24 h average, heart24, heart48 and heart72 models were statistically significant. All analyses were conducted using Stata 17.0 (StataCorp, College Station, TX) and R 4.3.1 (R Foundation for Statistical Computing, Vienna, Austria), with statistical significance defined as *p* < .05.

## 3. Results

### 3.1. Baseline of subjects

As shown in Table 1, 1257 participants were enrolled according to the predefined inclusion and exclusion criteria in this study. The average follow-up duration was 28.72 ± 21.14 months, with a mean age of 60.42 ± 14.19 years. The cohort comprised 1013 males (80.59%) and 244 females (19.41%). Among them, 1028 individuals did not experience MACEs after hospital discharge, whereas 229 did. Statistically significant differences were observed between the MACEs and non-MACEs groups concerning age, gender, diastolic blood pressure (DBP), smoking, drinking, hypertension, stroke, previous myocardial infarction, Killip classification, NT-proBNP, LDL, TGs, creatinine, expired myocardial infarction, left ventricular ejection fraction (LVEF), left circumflex artery (LCX), stenosis of other brunches, and inpatient days (*p* < .05).

**Table 1.** Baseline data of the subjects.

Variables	Total (*n* = 1257)	Group		Statistics	*p*	Missing data, *n* (%)
		None (*n* = 1028)	MACEs (*n* = 229)			
Age, mean ± SD	60.42 ± 14.19	59.20 ± 14.06	65.92 ± 13.49	*t* = −6.59	**<.001**	–
Gender, *n* (%)				*χ*^2^ = 27.82	**<.001**	–
Male	1013 (80.59)	857 (83.37)	156 (68.12)			
Female	244 (19.41)	171 (16.63)	73 (31.88)			
BMI, *M* (*Q*_1_, *Q*_3_)	24.28 (22.58, 26.26)	24.38 (22.74, 26.35)	23.89 (21.97, 26.04)	*Z* = −1.86	.063	227 (18.06)
SBP, mean ± SD	129.49 ± 22.14	129.78 ± 22.29	128.17 ± 21.44	*t* = 1.00	.318	–
DBP, mean ± SD	79.83 ± 15.02	80.31 ± 14.94	77.69 ± 15.20	*t* = 2.39	**.017**	–
Smoking, *n* (%)				*χ*^2^ = 18.97	**<.001**	–
No	605 (48.13)	465 (45.23)	140 (61.14)			
Yes	652 (51.87)	563 (54.77)	89 (38.86)			
Drinking, *n* (%)				*χ*^2^ = 13.40	**<.001**	–
No	940 (74.78)	747 (72.67)	193 (84.28)			
Yes	317 (25.22)	281 (27.33)	36 (15.72)			
Hypertension, *n* (%)				*χ*^2^ = 21.96	**<.001**	–
No	648 (51.55)	562 (54.67)	86 (37.55)			
Yes	609 (48.45)	466 (45.33)	143 (62.45)			
Diabetes, *n* (%)				*χ*^2^ = 3.08	.079	–
No	977 (77.72)	809 (78.70)	168 (73.36)			
Yes	280 (22.28)	219 (21.30)	61 (26.64)			
Stroke, *n* (%)				*χ*^2^ = 6.94	**.008**	–
No	1170 (93.08)	966 (93.97)	204 (89.08)			
Yes	87 (6.92)	62 (6.03)	25 (10.92)			
COPD, *n* (%)				*χ*^2^ = 0.61	.434	–
No	1208 (96.10)	990 (96.30)	218 (95.20)			
Yes	49 (3.90)	38 (3.70)	11 (4.80)			
Atrial fibrillation, *n* (%)				*χ*^2^ = 1.09	.297	1 (0.08)
No	1192 (94.83)	978 (95.14)	214 (93.45)			
Yes	65 (5.17)	50 (4.86)	15 (6.55)			
Tumour, *n* (%)				*χ*^2^ = 0.03	.867	–
No	1222 (97.22)	999 (97.18)	223 (97.38)			
Yes	35 (2.78)	29 (2.82)	6 (2.62)			
Old myocardial infarction, *n* (%)				*χ*^2^ = 14.48	**<.001**	–
No	1182 (94.03)	979 (95.23)	203 (88.65)			
Yes	75 (5.97)	49 (4.77)	26 (11.35)			
Myocardiopathy, *n* (%)				*χ*^2^ = 0.00	.982	–
No	1238 (98.49)	1013 (98.54)	225 (98.25)			
Yes	19 (1.51)	15 (1.46)	4 (1.75)			
Killip, *n* (%)				*χ*^2^ = 40.17	**<.001**	–
I	990 (78.76)	838 (81.52)	152 (66.38)			
II	156 (12.41)	121 (11.77)	35 (15.28)			
III	31 (2.47)	23 (2.24)	8 (3.49)			
IV	80 (6.36)	46 (4.47)	34 (14.85)			
NT-proBNP, *M* (*Q*_1_, *Q*_3_)	217.50 (65.00, 781.00)	171.70 (53.47, 619.69)	673.75 (159.70, 2855.00)	*Z* = −9.00	**<.001**	16 (1.27)
cTnT, *M* (*Q*_1_, *Q*_3_)	4.69 (0.39, 29.56)	4.67 (0.37, 29.17)	5.41 (0.47, 31.01)	*Z* = −0.33	.743	1 (0.08)
CK-MB, *M* (*Q*_1_, *Q*_3_)	81.83 (23.80, 198.60)	82.05 (23.90, 199.03)	78.40 (23.20, 194.00)	*Z* = −0.16	.873	101(8.04)
LDL, *M* (*Q*_1_, *Q*_3_)	2.66 (2.08, 3.22)	2.71 (2.14, 3.26)	2.48 (1.93, 3.02)	*Z* = −3.50	**<.001**	88 (7.00)
TG, *M* (*Q*_1_, *Q*_3_)	1.44 (1.00, 2.07)	1.49 (1.00, 2.15)	1.35 (0.95, 1.72)	*Z* = −2.88	**.004**	88 (7.00)
Creatine, *M* (*Q*_1_, *Q*_3_)	75.00 (64.00, 89.00)	74.00 (63.15, 88.00)	77.00 (65.00, 96.00)	*Z* = −2.29	**.022**	–
Expired myocardial infarction, *n* (%)				*χ*^2^ = 4.84	**.028**	–
No	898 (71.44)	748 (72.76)	150 (65.50)			
Yes	359 (28.56)	280 (27.24)	79 (34.50)			
LVEF, mean ± SD	54.98 ± 9.66	55.54 ± 9.27	52.46 ± 10.93	*t* = 3.97	**<.001**	26 (2.07)
LM, *n* (%)				*χ*^2^ = 3.45	.063	–
No	1176 (93.56)	968 (94.16)	208 (90.83)			
Yes	81 (6.44)	60 (5.84)	21 (9.17)			
LAD, *n* (%)				*χ*^2^ = 3.67	.055	–
No	170 (13.52)	148 (14.40)	22 (9.61)			
Yes	1087 (86.48)	880 (85.60)	207 (90.39)			
LCX, *n* (%)				*χ*^2^ = 16.41	**<.001**	–
No	529 (42.08)	460 (44.75)	69 (30.13)			
Yes	728 (57.92)	568 (55.25)	160 (69.87)			
RA, *n* (%)				*χ*^2^ = 1.63	.202	–
No	441 (35.08)	369 (35.89)	72 (31.44)			
Yes	816 (64.92)	659 (64.11)	157 (68.56)			
Other brunches, *n* (%)				*χ*^2^ = 27.73	**<.001**	–
No	764 (60.78)	660 (64.20)	104 (45.41)			
Yes	493 (39.22)	368 (35.80)	125 (54.59)			
βBlock, *n* (%)				*χ*^2^ = 1.32	.251	1 (0.08)
No	283 (22.51)	238 (23.15)	45 (19.65)			
Yes	974 (77.49)	790 (76.85)	184 (80.35)			
CCB, *n* (%)				*χ*^2^ = 2.08	.149	–
No	1193 (94.91)	980 (95.33)	213 (93.01)			
Yes	64 (5.09)	48 (4.67)	16 (6.99)			
ACEI/ARB, *n* (%)				*χ*^2^ = 0.82	.366	–
No	522 (41.53)	433 (42.12)	89 (38.86)			
Yes	735 (58.47)	595 (57.88)	140 (61.14)			
Inpatient days, *M* (*Q*_1_, *Q*_3_)	7.00 (5.00, 9.00)	7.00 (5.00, 9.00)	7.00 (5.00, 11.00)	*Z* = −2.63	**.009**	–
Survival time, mean ± SD	28.72 ± 21.14	33.02 ± 20.28	9.41 ± 12.29	*t* = 22.93	<.001	–

*χ*^2^: Chi-square test; *t*: *t*-test; *Z*: Mann–Whitney’s test; SD: standard deviation; *M*: median; *Q*_1_: 1st quartile; *Q*_3_: 3st quartile.

Missing data: including BMI, atrial fibrillation, NT-proBNP, cTnT, CK-MB, LDL, TG, LVEF and βBlock. A *p*-value of < 0.05 was considered to indicate a statistically significant difference between the groups.

### 3.2. Growth-building trajectory modelling

**Figure 2.**
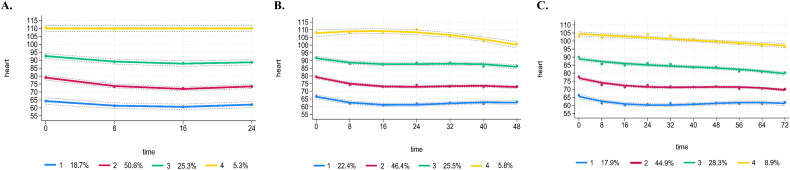
Trajectory model. (A) Modeling of the 24-hour heart rate trajectory. (B) Modeling of the 48-hour heart rate trajectory. (C) Modeling of the 72-hour heart rate trajectory.

For trajectory modelling, we initially fixed the polynomial degree to cubic and explored trajectory models with 1–5 groups. BIC and AIC values consistently decreased across models from 1 to 5. After evaluating entropy, AvePP, minimum sample size for trajectory groups, curve similarity, model simplicity and interpretability, we determined that the four-trajectory model (quadratic, quadratic, quadratic, intercept) provided the best fit for the 24-hour heart rate trajectory. The four-trajectory model (cubic, cubic, cubic and quadratic) was selected for the 48-hour heart rate trajectory, and the four-trajectory model (cubic, cubic, cubic and linear) for the 72-hour heart rate trajectory. Detailed modelling procedures can be found in the Supplement A1, and Figure 2 presents the results of the optimal trajectory models.

### 3.3. The Kaplan–Meier survival curve

**Figure 3.**
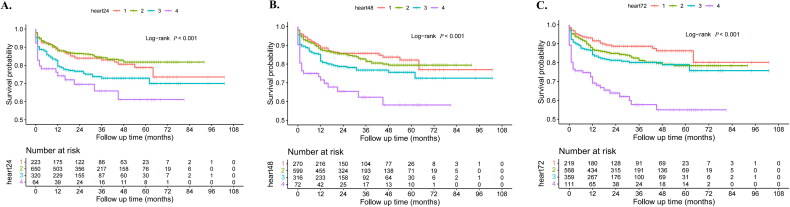
The Kaplan–Meier survival curve. (A) The K–M survival curve for the 24-hour heart rate trajectory. (B) The K–M survival curve for the 48-hour heart rate trajectory. (C) The K–M survival curve for the 72-hour heart rate trajectory.

Kaplan–Meier’s survival curves were employed to assess the cumulative incidence of MACEs after hospital discharge across heart rate trajectory groups at 24, 48 and 72 h. As illustrated in Figure 3, these survival curves were plotted and analysed using Log-rank tests. The analysis revealed that the incidence of MACEs in group 1 was significantly lower than that in groups 3 and 4 across all time points (Log-rank *p* < .001).

### 3.4. Multivariate Cox regression analysis

Multivariate Cox regression analysis as shown in Table 2 revealed that, each unit increase in the 24-hour average heart rate was associated with an increased hazard risk of MACEs after hospital discharge in STEMI patients (aHR = 1.016, 95% CI: 1.003–1.030, *p* = .015). In the 48-hour heart rate trajectory model, trajectory 4 was associated with a significantly higher hazard risk of MACEs compared to trajectory 1 (aHR = 2.163, 95% CI: 1.204–3.885, *p* = .009). In the 72-hour heart rate trajectory model, trajectory 3 was associated with a significantly lower hazard risk of MACEs compared to trajectory 1 (aHR = 0.669, 95% CI: 0.461–0.976, *p* = .040), while trajectory 4 was associated with a significantly higher hazard risk of MACEs compared to trajectory 1 (aHR = 3.195, 95% CI: 1.813–5.632, *p* < .001).

**Table 2.** Multivariate Cox regression analysis.

Variables	Model 1		Model 2		Model 3	
	HR (95% CI)	*p*	HR (95% CI)	*p*	HR (95% CI)	*p*
24 h average	1.028 (1.016–1.040)	**<.001**	1.028 (1.016–1.040)	**<.001**	1.016 (1.003–1.030)	**.015**
Heart24						
1	1.00 (reference)		1.00 (reference)		1.00 (reference)	
2	0.869(0.596–1.266)	.463	0.983 (0.673–1.435)	.928	0.860 (0.577–1.280)	.457
3	1.553 (1.053–2.291)	**.026**	1.779 (1.202–2.633)	**.004**	1.413 (0.921–2.169)	.113
4	2.217 (1.290–3.812)	**.004**	2.690 (1.558–4.647)	**<.001**	1.816 (0.987–3.340)	.054
Heart48						
1	1.00 (reference)		1.00 (reference)		1.00 (reference)	
2	1.126 (0.779–1.628)	.528	1.228 (0.848–1.778)	.277	1.099 (0.743–1.626)	.635
3	1.586 (1.073–2.345)	**.021**	1.746 (1.179–2.585)	**.005**	1.358 (0.881–2.093)	.164
4	2.954 (1.791–4.870)	**<.001**	3.334 (2.015–5.517)	**<.001**	2.163 (1.204–3.885)	**.009**
Heart72						
1	1.00 (reference)		1.00 (reference)		1.00 (reference)	
2	1.488 (0.964–2.296)	.073	1.577 (1.020–2.437)	**.040**	1.535 (0.974–2.419)	.064
3	1.752 (1.114–2.756)	**.015**	1.938 (1.229–3.057)	**.004**	1.669 (1.022–2.727)	**.040**
4	4.053 (2.478–6.629)	**<.001**	4.058 (2.472–6.660)	**<.001**	3.195 (1.813–5.632)	**<.001**

Model 1: unadjusted model. Model 2: adjusted for age, gender and BMI. Model 3: adjusted for SBP, DBP, smoking, drinking, hypertension, COPD, atrial fibrillation, tumour, myocardiopathy, diabetes, stroke, old myocardial infarction, Killip class, NT-proBNP, LDL, TG, creatinine, expired myocardial infarction, LVEF, LM, LAD, LCX, RA, other branches, inpatient days, cTnT, CK-MB, βBlock, CCB and ACEI/ARB in addition to the variables in model 2. A *p*-value of < 0.05 was considered to indicate a statistically significant difference between the groups.

### 3.5. Subgroup analysis

**Figure 4.**
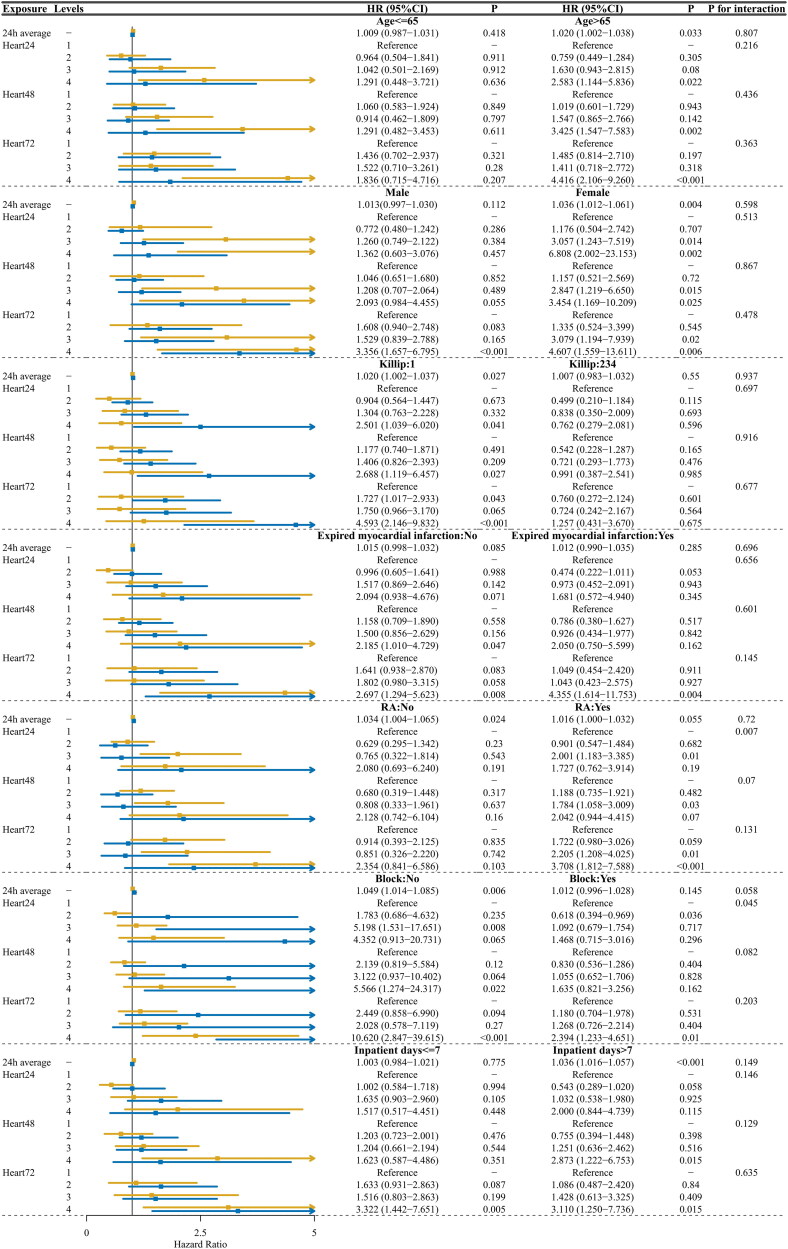
Subgroup analysis and forest plot.

Subgroup analysis (Figure 4 and Supplement A2) based on various factors including age (with a threshold of 65 years), gender, Killip classification, presence of previous myocardial infarction, RA occlusion, use of β-blockers, and length of inpatient stay (with a threshold of seven days).

Subgroup analysis of the 24-hour heart rate trajectory model revealed significant heterogeneity in the association between heart rate trajectories and outcomes across different subpopulations. Specifically, we observed a significant interaction between 24-hour heart rate trajectory and RA (*p* for interaction = .007), as well as β-blocker use (*p* for interaction = .045).

### 3.6. Predictive value of trajectories

**Figure 5.**
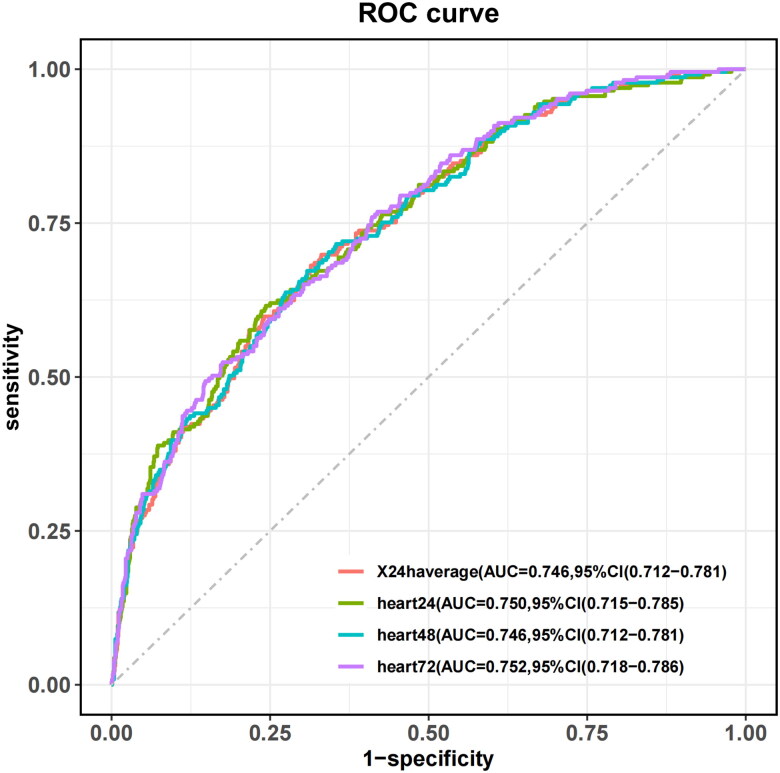
ROC for trajectories.

The results of the DeLong test (Figure 5 and Supplement A3) indicated no statistically significant differences in the AUCs among the models: 24 h average (AUC = 0.741, 95% CI: 0.704–0.777), heart24 (AUC = 0.743, 95% CI: 0.706–0.781), heart48 (AUC = 0.741, 95% CI: 0.704–0.777) and heart72 (AUC = 0.745, 95% CI: 0.709–0.781) (*p* > .05).

## 4. Discussion

This study focuses on investigating the early changes in heart rate trajectories in STEMI patients following appropriate and standardized PCI treatment. By employing GBTM, we identified distinct patterns of heart rate trajectory changes at various time points post-PCI. These trajectory patterns exhibited different clinical characteristics, with higher heart rate trajectory groups being significantly associated with MACEs after hospital discharge.

Heart rate is a crucial determinant of myocardial oxygen consumption in CAD patients, and extensive research has established a correlation between heart rate and the prognosis of AMI. Our study further confirms that elevated heart rate levels are associated with MACEs, consistent with previous research [22]. The well-known GRACE risk score considers heart rate as an independent risk factor for assessing the prognosis of patients with AMI [23]. Current research has overlooked the dynamic nature of heart rate, typically focusing on heart rate measurements taken at admission, pre-PCI, or at discharge to correlate with the incidence of MACEs [11,12]. It remains unclear whether heart rate trajectory changes might provide a better reflection of prognosis in STEMI patients. The 72-hour period post-myocardial infarction is considered the golden phase for early ventricular remodelling [20]. Despite effective reperfusion therapy reducing myocardial infarction size, the heart undergoes a transition from acute injury to chronic remodelling during this stage, with processes such as myocardial apoptosis, inflammation and fibrosis gradually initiating [23–25]. Myocardial self-repair inevitably triggers associated immune or inflammatory responses (including vasopressin systems, the renin–angiotensin–aldosterone system (RAAS) and adrenergic activation), which mediate ventricular remodelling [26–28]. Abnormal ventricular dilation or myocardial fibrosis, potentially accompanied by impaired myocardial biomechanics, mitochondrial dysfunction or oxidative stress, may lead to inappropriate increases or decreases in heart rate [29]. But this procedure of myocardial repairment is disadvantageous as it modifies the duration of the myocardial action potential. Simultaneously, electrical remodelling of the myocardium is also advancing. Variations in heart rate can significantly affect the electrophysiological properties of the myocardium, including the duration of the action potential and the repolarization process. Increased heart rates generally accelerate myocardial electrical remodelling, leading to a reduction in action potential duration and alterations in repolarization. These changes may consequently elevate the risk of arrhythmias. And heart rate can indirectly reflect the activity of the sympathetic or parasympathetic nervous systems, and to some extent, it can directly or indirectly impact coronary blood supply [30–32].

For patients with myocardial infarction, heart rate trajectories can reflect the autonomic nervous system’s response to acute stressors [18,33]. In our study, we identified distinct heart rate trajectory patterns in STEMI patients during the short-term post-PCI period. After adjusting for confounding factors, we found no statistically significant association between the different heart rate trajectory groups within 24 h and the occurrence of MACEs after hospital discharge. However, over time, higher heart rate trajectories were associated with poorer long-term outcomes. Current guidelines recommend maintaining a heart rate of 55–60 beats per minute (bpm) in post-myocardial infarction patients [2]. As previously noted, STEMI patients may be influenced by stress and sympathetic nervous system activation, which could impair short-term heart rate control. This period coincides with a critical phase of ventricular remodelling. After adjusting for relevant confounders, we observed no statistically significant differences in long-term outcomes for the 24-hour trajectory group 2 (70–80 bpm), 48-hour trajectory group 2 (72–80 bpm) and 72-hour trajectory group 2 (70–77 bpm) in relation to MACEs after hospital discharge. This suggests that strict adherence to the guideline-recommended heart rate in the immediate post-PCI period may not be necessary for STEMI patients. Observing the trajectory patterns, we found that a short-term heart rate of 60–77 bpm was acceptable, which aligns with the principle of gradual dose titration used in beta-blocker therapy, where small doses are adjusted until the patient can tolerate them. In the 48-hour trajectory pattern analysis, we found no significant difference in long-term after hospital discharge MACEs between trajectory groups 2 (72–80 bpm) and 3 (85–93 bpm) compared to trajectory group 1 (60–67 bpm). However, in the 72-hour trajectory pattern, we observed a 0.669-fold increase in the hazard of MACEs in trajectory group 3 (80–90 bpm) compared to trajectory group 1, with statistical significance. This suggests that heart rate control below 80 bpm by day 3 post-PCI may be associated with better long-term outcomes. For STEMI patients with a heart rate >80 bpm in the short-term, the hazard risk of long-term after hospital discharge MACEs significantly increases. This may be attributed to the acute stress state following myocardial infarction, which leads to autonomic imbalance and increased risk of MACEs. Elevated heart rates in the short-term may contribute to increased coronary pressure, arterial stiffness and endothelial damage, as well as exacerbate myocardial ischemia and impair ventricular remodelling, thereby increasing the risk of adverse outcomes [34–36]. Sustained high heart rate trajectories are associated with increased myocardial oxygen demand, shortened diastolic duration and reduced myocardial perfusion, all of which can lead to myocardial cell apoptosis and, ultimately, fibrosis. This process may represent an early stage in the progression toward ventricular remodelling. In contrast, low heart rate trajectories allow for more ample perfusion filling time, which may effectively mitigate myocardial hypoxia and attenuate myocardial fibrosis. These findings are consistent with the observations of Raby et al. who reported that patients with elevated heart rates postoperatively had higher catecholamine levels, likely accelerating adverse ventricular remodelling [37]. However, these explanations are based on existing literature and our cohort study results, and the underlying cellular and molecular mechanisms require further exploration. Although the predictive value of final heart rate trajectory patterns for MACEs is limited, early heart rate trajectory patterns provide valuable clinical insights. These patterns offer higher clinical value compared to the single-point 24-hour average heart rate, providing a more comprehensive understanding of patient prognosis.

In the subgroup analysis, we observed that patients over 65 years old with a higher heart rate trajectory were more prone to MACEs. This susceptibility may be linked to the reduced self-repair capacity of elderly myocardial infarction patients, who struggle to correct maladaptive stress responses, thus accelerating the fibrosis of infarcted areas [38,39]. Aging is associated with decreased vascular wall elasticity and increased arterial stiffness, which can alter haemodynamics and indirectly affect heart rate [40,41]. Our study indicates that a higher heart rate trajectory correlates with poorer cardiovascular outcomes, highlighting the importance of early heart rate management in this demographic. Regarding gender, both males and females exhibiting a high heart rate trajectory within the first 72 h post-MI were identified as high-risk for MACEs. In women, sensitivity to higher heart rate trajectories was observed as early as 24 h post-myocardial infarction. This may be due to the stress response associated with myocardial infarction, which could make women more sensitive to corticotropin-releasing factor (CRF) [42]. CRF influences the norepinephrine system in the brain, leading to heightened sympathetic nervous system activation. These findings underscore the critical importance of heart rate control in the female population. In patients without heart failure (Killip class I), a significant association between high heart rate trajectory and MACEs after hospital discharge was observed. Conversely, in patients with MI complicated by heart failure, this association was not evident, potentially due to sample size limitations leading to bias. The influence of compensatory heart rate elevation on prognosis in STEMI patients with heart failure was not apparent in this study, and the effect of PCI on the progression of cardiac dysfunction in heart failure patients remains uncertain, warranting further research. For patients with chronic MI who undergo timely PCI, managing heart rate is crucial. Chronic MI induces a prolonged self-repair state, and reducing heart rate may help mitigate adverse ventricular remodelling. Nonetheless, timely reperfusion therapy remains essential for this patient population. We identified an interaction between the 24-hour heart rate trajectory and RA stenosis. This finding is understandable given that the majority of pacemaker cells are supplied by the RA. Among patients with RA stenosis, a higher early heart rate trajectory predicted worse outcomes and a significantly increased risk compared to those with a lower heart rate trajectory, likely due to compensatory increases in ectopic pacemaker activity [43]. Concerning the short-term use of beta-blockers, an interaction with the 24-hour heart rate trajectory was observed. Beta-blocker therapy indeed improved outcomes in patients with a higher heart rate trajectory, suggesting a beneficial role in managing such cases.

This study inevitably has several limitations. First, the heart rate trajectory model is data-driven, and some of the potential grouping relationships remain unexplained. We focused exclusively on heart rate changes during the early phase of ventricular remodelling within the first 72 h, leaving the effects of heart rate variations during the entire hospital stay and over long-term follow-up on myocardial infarction patient outcomes unclear. Furthermore, it is not possible to rule out post-MI malignant arrhythmic events based solely on heart rate trajectories. Our study also excluded patients with severe comorbid conditions; therefore, the trajectory models cannot explain the effects in this population. Second, as our study is based on a retrospective cohort, the causal relationship between heart rate trajectories and MI outcomes remains uncertain. In classifying heart failure among MI patients, we employed the Killip classification; however, further stratified analyses based on LVEF were not conducted. As a result, we cannot distinguish whether the accelerated heart rate is primarily due to sympathetic activation following acute MI or whether it is a compensatory response to declining cardiac function. Current guidelines recommend titrating beta-blockers at low doses, yet we were unable to assess the impact of dosing on heart rate trajectory interventions. While we observed an interaction between beta-blocker use and heart rate trajectory over 24 h, its effect on outcomes within the first 72 h remains unclear. This ambiguity may be due to the limited sample size or the short recording period for heart rate trajectory changes. Although subgroup analyses in our study revealed heterogeneity between groups, we acknowledge that due to the nature of retrospective cohort studies, which are limited by sample size and the challenges of establishing causal relationships, we should be cautious in overemphasizing the heterogeneity of heart rate trajectories across subgroups in the interpretation of our results. Due to variations in Holter monitoring equipment, we were unable to comprehensively collect HRV parameters, such as the standard deviation of normal-to-normal (SDNN) intervals or the root mean square of successive differences (RMSSDs). Therefore, the relationship between heart rate trajectory and the quality of dynamic electrocardiographic monitoring remains undetermined. Finally, it is important to acknowledge that, as an exploratory study, the heart rate trajectories within the first 72 h provide valuable clinical insights and guidance, highlighting the significance of heart rate management. However, their predictive value for long-term MACEs remains limited, comparable to that of the 24-hour average heart rate. Future research should consider utilizing more advanced tools (such as electronic wearables) or more refined methodologies to enhance heart rate management and monitoring in post-PCI STEMI patients.

## 5. Conclusions

We examined heart rate trajectories during the early phase of ventricular remodelling (within the first 72 h) in patients with STEMI who underwent PCI. Elevated heart rate trajectories with rates greater than 80 bpm within 72 h post-PCI are associated with an increased risk of MACEs after hospital discharge. Heart rate management should be further emphasized in post-PCI STEMI patients.

## Supplementary Material

Supplemental Material.docx

## Data Availability

The derived data that were generated in the current study are available from the corresponding author upon reasonable request.
