# Supplementary material for: Association between early heart rate trajectories in post-PCI STEMI patients and prognosis after hospital discharge
Source: Ann Med. 2025 Feb 22;57(1):2468267. doi: 10.1080/07853890.2025.2468267 (PMC11849009; doi:10.1080/07853890.2025.2468267)
Supplement: Supplemental Material.docx [file IANN_A_2468267_SM7959.docx]

**Supplement**

**Abbreviations**

**AIC: Akaike’s information criterion**

**AvePP: average posterior probabilities**

**BIC: Bayesian information criterion**

**MACE: Major Adverse Cardiovascular Events**

**LDL: Low-Density Lipoprotein**

**TG: Triglycerides**

**SBP: Systolic Blood Pressure**

**DBP: Diastolic Blood Pressure**

**LVEF: Left Ventricular Ejection Fraction**

**LM: Left Main (Coronary Artery)**

**LAD: Left Anterior Descending (Coronary Artery)**

**LCX: Left Circumflex (Coronary Artery)**

**A1 The fitting of trajectory**

1. **24h heart rate trajectories**

**Table S1 Model Fitting of 24-Hour Heart Rate Trajectories**

| Model Fit Metrics | Number of classes | | | | |
| --- | --- | --- | --- | --- | --- |
|  | 1 | 2 | 3 | 4 | 5 |
| BIC | -20888.31 | -20095.02 | -19826.41 | -19714.68 | -19677.67 |
| AIC | -20875.47 | -20069.34 | -19787.89 | -19663.32 | -19613.46 |
| Entropy |  | 0.837 | 0.817 | 0.785 | 0.791 |
| Class proportion, % |  |  |  |  |  |
| Class1 |  | 72.30 | 52.40 | 18.91 | 13.61 |
| Class2 |  | 27.70 | 39.57 | 50.59 | 47.76 |
| Class3 |  |  | 8.03 | 25.19 | 28.09 |
| Class4 |  |  |  | 5.31 | 8.65 |
| Class5 |  |  |  |  | 1.89 |
| AvePP |  |  |  |  |  |
| Class1 |  | 0.96 | 0.93 | 0.85 | 0.85 |
| Class2 |  | 0.92 | 0.90 | 0.87 | 0.86 |
| Class3 |  |  | 0.94 | 0.87 | 0.85 |
| Class4 |  |  |  | 0.95 | 0.87 |
| Class5 |  |  |  |  | 0.92 |

Abbreviation: BIC: Bayesian information criterion, AIC: Akaike’s information criterion, AvePP: average posterior probabilities

**Table S2 Estimation of the 4-Trajectory Model for 24-Hour Heart Rate**

| Class | Parameter | Estimate | Standard Error | T for H0:  Parameter=0 | Prob > \|T\| |
| --- | --- | --- | --- | --- | --- |
| 1 | Intercept | 64.25 | 1.03 | 62.04 | 0.00 |
|  | Linear | -0.49 | 0.14 | -3.55 | 0.00 |
|  | Quadratic | 0.01 | 0.00 | 3.13 | 0.00 |
| 2 | Intercept | 79.01 | 0.76 | 103.72 | 0.00 |
|  | Linear | -0.87 | 0.76 | -10.28 | 0.00 |
|  | Quadratic | 0.02 | 0.00 | 8.22 | 0.00 |
| 3 | Intercept | 92.58 | 0.80 | 115.65 | 0.00 |
|  | Linear | -0.56 | 0.12 | -4.44 | 0.00 |
|  | Quadratic | 0.01 | 0.00 | 3.47 | 0.00 |
| 4 | Intercept | 109.95 | 1.00 | 109.37 | 0.00 |

Based on **Tables S1 and S2**, we can ascertain that throughout Models 1 to 5, both BIC and AIC values showed a consistent decline. After considering Entropy, Average Posterior Probability (AvePP), minimum sample size for trajectory groups, curve similarity, model simplicity, and interpretability, the optimal fit for the 24-hour heart rate trajectory was achieved with a 4-trajectory model (quadratic, quadratic, quadratic, intercept).

1. **48h heart rate trajectories**

**Table S3 Model Fitting of 48-Hour Heart Rate Trajectories**

| Model Fit Metrics | Number of classes | | | | |
| --- | --- | --- | --- | --- | --- |
|  | 1 | 2 | 3 | 4 | 5 |
| BIC | -36243.55 | -34478.19 | -33859.58 | -33594.30 | -33457.08 |
| AIC | -36230.71 | -34442.78 | -33821.05 | -33542.94 | -33392.87 |
| Entropy | / | 0.894 | 0.882 | 0.854 | 0.855 |
| Class proportion, % |  |  |  |  |  |
| Class1 | 1 | 68.36 | 49.42 | 22.37 | 16.52 |
| Class2 |  | 31.64 | 39.25 | 46.39 | 42.12 |
| Class3 |  |  | 11.33 | 25.49 | 28.59 |
| Class4 |  |  |  | 5.75 | 10.76 |
| Class5 |  |  |  |  | 2.00 |
| AvePP |  |  |  |  |  |
| Class1 | 1 | 0.97 | 0.95 | 0.92 | 0.89 |
| Class2 |  | 0.96 | 0.94 | 0.90 | 0.91 |
| Class3 |  |  | 0.95 | 0.93 | 0.89 |
| Class4 |  |  |  | 0.96 | 0.94 |
| Class5 |  |  |  |  | 0.94 |

Abbreviation: BIC: Bayesian information criterion, AIC: Akaike’s information criterion, AvePP: average posterior probabilities

**Table S4 Estimation of the 4-Trajectory Model for 48-Hour Heart Rate**

| Class | Parameter | Estimate | Standard Error | T for H0:  Parameter=0 | Prob > \|T\| |
| --- | --- | --- | --- | --- | --- |
| 1 | Intercept | 66.45 | 0.70 | 93.98 | 0.00 |
|  | Linear | -0.65 | 0.11 | -5.69 | 0.00 |
|  | Quadratic | 0.02 | 0.00 | 4.28 | 0.00 |
|  | Cubic | -0.00 | 0.00 | -3.38 | 0.00 |
| 2 | Intercept | 79.17 | 0.53 | 147.18 | 0.00 |
|  | Linear | -0.71 | 0.08 | -8.93 | 0.00 |
|  | Quadratic | 0.02 | 0.00 | 6.40 | 0.00 |
|  | Cubic | -0.00 | 0.00 | -5.20 | 0.00 |
| 3 | Intercept | 91.46 | 0.61 | 148.43 | 0.00 |
|  | Linear | -0.49 | 0.11 | -4.46 | 0.00 |
|  | Quadratic | 0.02 | 0.00 | 3.66 | 0.00 |
|  | Cubic | -0.00 | 0.00 | -3.43 | 0.00 |
| 4 | Intercept | 107.82 | 1.16 | 92.60 | 0.00 |
|  | Linear | 0.20 | 0.10 | 1.96 | 0.04 |
|  | Quadratic | -0.01 | 0.00 | -3.70 | 0.00 |

In **Table S3**, a 4-trajectory model was selected for the 48-hour data fitting based on BIC and AIC criteria, alongside considerations of Entropy and AvePP. **Table S4** demonstrates that the 4-trajectory model (cubic, cubic, cubic, quadratic) provided the best fit.

1. **72h heart rate trajectories**

**Table S5 Model Fitting of 72-Hour Heart Rate Trajectories**

| Model Fit Metrics | Number of classes | | | | |
| --- | --- | --- | --- | --- | --- |
|  | 1 | 2 | 3 | 4 | 5 |
| BIC | -51323.13 | -48649.51 | -47702.16 | -47299.54 | -47040.02 |
| AIC | -51310.29 | -48623.83 | -47663.64 | -47248.18 | -46975.81 |
| Entropy | / | 0.921 | 0.909 | 0.874 | 0.888 |
| Class proportion, % |  |  |  |  |  |
| Class1 | 1 | 68.09 | 49.59 | 18.01 | 14.59 |
| Class2 |  | 31.91 | 39.94 | 44.88 | 42.43 |
| Class3 |  |  | 11.46 | 28.29 | 29.02 |
| Class4 |  |  |  | 8.82 | 12.06 |
| Class5 |  |  |  |  | 1.90 |
| AvePP |  |  |  |  |  |
| Class1 | 1 | 0.98 | 0.97 | 0.92 | 0.91 |
| Class2 |  | 0.97 | 0.94 | 0.93 | 0.93 |
| Class3 |  |  | 0.97 | 0.93 | 0.93 |
| Class4 |  |  |  | 0.96 | 0.94 |
| Class5 |  |  |  |  | 0.96 |

Abbreviation: BIC: Bayesian information criterion, AIC: Akaike’s information criterion, AvePP: average posterior probabilities

**Table S6 Estimation of the 4-Trajectory Model for 72-Hour Heart Rate**

| Class | Parameter | Estimate | Standard Error | T for H0:  Parameter=0 | Prob > \|T\| |
| --- | --- | --- | --- | --- | --- |
| 1 | Intercept | 65.49 | 0.69 | 94.08 | 0.00 |
|  | Linear | -0.45 | 0.07 | -5.82 | 0.00 |
|  | Quadratic | 0.01 | 0.00 | 4.69 | 0.00 |
|  | Cubic | -0.00 | 0.00 | -3.90 | 0.00 |
| 2 | Intercept | 76.94 | 0.52 | 146.91 | 0.00 |
|  | Linear | -0.44 | 0.04 | -9.08 | 0.00 |
|  | Quadratic | 0.011 | 0.00 | 6.92 | 0.00 |
|  | Cubic | -0.00 | 0.00 | -6.09 | 0.00 |
| 3 | Intercept | 88.86 | 0.55 | 160.66 | 0.00 |
|  | Linear | -0.24 | 0.06 | -3.87 | 0.00 |
|  | Quadratic | 0.00 | 0.00 | 2.22 | 0.02 |
|  | Cubic | -0.00 | 0.00 | -2.23 | 0.02 |
| 4 | Intercept | 104.45 | 0.64 | 161.25 | 0.00 |
|  | Linear | -0.10 | 0.01 | -7.84 | 0.00 |

**Table S5** integrates BIC and AIC criteria, along with Entropy, AvePP, and the minimum sample size for trajectory groups, leading to the selection of a 4-trajectory model. As shown in **Table S6**, the 72-hour heart rate trajectory was best represented by a 4-trajectory model (cubic, cubic, cubic, linear).

## A2. Subgroup Analysis

Table S7. Subgroup Analysis

| **Subgroup** | **Exposure** | **Levels** | HR (95%CI) | P | P for interaction | |
| --- | --- | --- | --- | --- | --- | --- |
| **Age** |  |  |  |  | |  |
| <=65  (n=787) | 24h average |  | 1.009 (0.987 ~ 1.031) | 0.418 | | 0.807 |
|  | Heart24 | 1 | Reference |  | | 0.216 |
|  |  | 2 | 0.964 (0.504 ~ 1.841) | 0.911 | |  |
|  |  | 3 | 1.042 (0.501 ~ 2.169) | 0.912 | |  |
|  |  | 4 | 1.291 (0.448 ~ 3.721) | 0.636 | |  |
|  | Heart48 | 1 | Reference |  | | 0.436 |
|  |  | 2 | 1.060 (0.583 ~ 1.924) | 0.849 | |  |
|  |  | 3 | 0.914 (0.462 ~ 1.809) | 0.797 | |  |
|  |  | 4 | 1.291 (0.482 ~ 3.453) | 0.611 | |  |
|  | Heart72 | 1 | Reference |  | | 0.363 |
|  |  | 2 | 1.436 (0.702 ~ 2.937) | 0.321 | |  |
|  |  | 3 | 1.522 (0.710 ~ 3.261) | 0.280 | |  |
|  |  | 4 | 1.836 (0.715 ~ 4.716) | 0.207 | |  |
| >65  (n=470) | 24h average |  | 1.020 (1.002 ~ 1.038) | 0.033 | |  |
|  | Heart24 | 1 | Reference |  | |  |
|  |  | 2 | 0.759 (0.449 ~ 1.284) | 0.305 | |  |
|  |  | 3 | 1.630 (0.943 ~ 2.815) | 0.080 | |  |
|  |  | 4 | 2.583 (1.144 ~ 5.836) | 0.022 | |  |
|  | Heart48 | 1 | Reference |  | |  |
|  |  | 2 | 1.019 (0.601 ~ 1.729) | 0.943 | |  |
|  |  | 3 | 1.547 (0.865 ~ 2.766) | 0.142 | |  |
|  |  | 4 | 3.425 (1.547 ~ 7.583) | 0.002 | |  |
|  | Heart72 | 1 | Reference |  | |  |
|  |  | 2 | 1.485 (0.814 ~ 2.710) | 0.197 | |  |
|  |  | 3 | 1.411 (0.718 ~ 2.772) | 0.318 | |  |
|  |  | 4 | 4.416 (2.106 ~ 9.260) | <0.001 | |  |
| Male  (n=1013) | 24h average |  | 1.013(0.997~1.030) | 0.112 | | 0.598 |
|  | Heart24 | 1 | Reference |  | | 0.513 |
|  |  | 2 | 0.772 (0.480 ~ 1.242) | 0.286 | |  |
|  |  | 3 | 1.260 (0.749 ~ 2.122) | 0.384 | |  |
|  |  | 4 | 1.362 (0.603 ~ 3.076) | 0.457 | |  |
|  | Heart48 | 1 | Reference |  | | 0.867 |
|  |  | 2 | 1.046 (0.651 ~ 1.680) | 0.852 | |  |
|  |  | 3 | 1.208 (0.707 ~ 2.064) | 0.489 | |  |
|  |  | 4 | 2.093 (0.984 ~ 4.455) | 0.055 | |  |
|  | Heart72 | 1 | Reference |  | | 0.478 |
|  |  | 2 | 1.608 (0.940 ~ 2.748) | 0.083 | |  |
|  |  | 3 | 1.529 (0.839 ~ 2.788) | 0.165 | |  |
|  |  | 4 | 3.356 (1.657 ~ 6.795) | <0.001 | |  |
| Female  (n=244) | 24h average |  | 1.036 (1.012~1.061) | 0.004 | |  |
|  | Heart24 | 1 | Reference |  | |  |
|  |  | 2 | 1.176 (0.504 ~ 2.742) | 0.707 | |  |
|  |  | 3 | 3.057 (1.243 ~ 7.519) | 0.014 | |  |
|  |  | 4 | 6.808 (2.002 ~23.153) | 0.002 | |  |
|  | Heart48 | 1 | Reference |  | |  |
|  |  | 2 | 1.157 (0.521 ~ 2.569) | 0.720 | |  |
|  |  | 3 | 2.847 (1.219 ~ 6.650) | 0.015 | |  |
|  |  | 4 | 3.454 (1.169 ~ 10.209) | 0.025 | |  |
|  | Heart72 | 1 | Reference |  | |  |
|  |  | 2 | 1.335 (0.524 ~ 3.399) | 0.545 | |  |
|  |  | 3 | 3.079 (1.194 ~ 7.939) | 0.020 | |  |
|  |  | 4 | 4.607 (1.559 ~ 13.611) | 0.006 | |  |
| **Killip** |  |  |  |  | |  |
| I (n=990) | 24h average |  | 1.020 (1.002 ~ 1.037) | 0.027 | | 0.937 |
|  | Heart24 | 1 | Reference |  | | 0.697 |
|  |  | 2 | 0.904 (0.564 ~ 1.447) | 0.673 | |  |
|  |  | 3 | 1.304 (0.763 ~ 2.228) | 0.332 | |  |
|  |  | 4 | 2.501 (1.039 ~ 6.020) | 0.041 | |  |
|  | Heart48 | 1 | Reference |  | | 0.916 |
|  |  | 2 | 1.177 (0.740 ~ 1.871) | 0.491 | |  |
|  |  | 3 | 1.406 (0.826 ~ 2.393) | 0.209 | |  |
|  |  | 4 | 2.688 (1.119 ~ 6.457) | 0.027 | |  |
|  | Heart72 | 1 | Reference |  | | 0.677 |
|  |  | 2 | 1.727 (1.017 ~ 2.933) | 0.043 | |  |
|  |  | 3 | 1.750 (0.966 ~ 3.170) | 0.065 | |  |
|  |  | 4 | 4.593 (2.146 ~ 9.832) | <0.001 | |  |
| II/III/IV (n=267) | 24h average |  | 1.007 (0.983 ~ 1.032) | 0.550 | |  |
|  | Heart24 | 1 | Reference |  | |  |
|  |  | 2 | 0.499 (0.210 ~ 1.184) | 0.115 | |  |
|  |  | 3 | 0.838 (0.350 ~ 2.009) | 0.693 | |  |
|  |  | 4 | 0.762 (0.279 ~ 2.081) | 0.596 | |  |
|  | Heart48 | 1 | Reference |  | |  |
|  |  | 2 | 0.542 (0.228 ~ 1.287) | 0.165 | |  |
|  |  | 3 | 0.721 (0.293 ~ 1.773) | 0.476 | |  |
|  |  | 4 | 0.991 (0.387 ~ 2.541) | 0.985 | |  |
|  | Heart72 | 1 | Reference |  | |  |
|  |  | 2 | 0.760 (0.272 ~ 2.124) | 0.601 | |  |
|  |  | 3 | 0.724 (0.242 ~ 2.167) | 0.564 | |  |
|  |  | 4 | 1.257 (0.431 ~ 3.670) | 0.675 | |  |
| **Expired myocardial infarction** |  |  |  |  | |  |
| No (n=898) | 24h average |  | 1.015 (0.998 ~ 1.032) | 0.085 | | 0.696 |
|  | Heart24 | 1 | Reference |  | | 0.656 |
|  |  | 2 | 0.996 (0.605 ~ 1.641) | 0.988 | |  |
|  |  | 3 | 1.517 (0.869 ~ 2.646) | 0.142 | |  |
|  |  | 4 | 2.094 (0.938 ~ 4.676) | 0.071 | |  |
|  | Heart48 | 1 | Reference |  | | 0.601 |
|  |  | 2 | 1.158 (0.709 ~ 1.890) | 0.558 | |  |
|  |  | 3 | 1.500 (0.856 ~ 2.629) | 0.156 | |  |
|  |  | 4 | 2.185 (1.010 ~ 4.729) | 0.047 | |  |
|  | Heart72 | 1 | Reference |  | | 0.145 |
|  |  | 2 | 1.641 (0.938 ~ 2.870) | 0.083 | |  |
|  |  | 3 | 1.802 (0.980 ~ 3.315) | 0.058 | |  |
|  |  | 4 | 2.697 (1.294 ~ 5.623) | 0.008 | |  |
| Yes (n=359) | 24h average |  | 1.012 (0.990 ~ 1.035) | 0.285 | |  |
|  | Heart24 | 1 | Reference |  | |  |
|  |  | 2 | 0.474 (0.222 ~ 1.011) | 0.053 | |  |
|  |  | 3 | 0.973 (0.452 ~ 2.091) | 0.943 | |  |
|  |  | 4 | 1.681 (0.572 ~ 4.940) | 0.345 | |  |
|  | Heart48 | 1 | Reference |  | |  |
|  |  | 2 | 0.786 (0.380 ~ 1.627) | 0.517 | |  |
|  |  | 3 | 0.926 (0.434 ~ 1.977) | 0.842 | |  |
|  |  | 4 | 2.050 (0.750 ~ 5.599) | 0.162 | |  |
|  | Heart72 | 1 | Reference |  | |  |
|  |  | 2 | 1.049 (0.454 ~ 2.420) | 0.911 | |  |
|  |  | 3 | 1.043 (0.423 ~ 2.575) | 0.927 | |  |
|  |  | 4 | 4.355 (1.614 ~ 11.753) | 0.004 | |  |
| **RA** |  |  |  |  | |  |
| No (n=441) | 24h average |  | 1.034 (1.004 ~ 1.065) | 0.024 | | 0.720 |
|  | Heart24 | 1 | Reference |  | | 0.007 |
|  |  | 2 | 0.629 (0.295 ~ 1.342) | 0.230 | |  |
|  |  | 3 | 0.765 (0.322 ~ 1.814) | 0.543 | |  |
|  |  | 4 | 2.080 (0.693 ~ 6.240) | 0.191 | |  |
|  | Heart48 | 1 | Reference |  | | 0.070 |
|  |  | 2 | 0.680 (0.319 ~ 1.448) | 0.317 | |  |
|  |  | 3 | 0.808 (0.333 ~ 1.961) | 0.637 | |  |
|  |  | 4 | 2.128 (0.742 ~ 6.104) | 0.160 | |  |
|  | Heart72 | 1 | Reference |  | | 0.131 |
|  |  | 2 | 0.914 (0.393 ~ 2.125) | 0.835 | |  |
|  |  | 3 | 0.851 (0.326 ~ 2.220) | 0.742 | |  |
|  |  | 4 | 2.354 (0.841 ~ 6.586) | 0.103 | |  |
| Yes (n=816) | 24h average |  | 1.016 (1.000 ~ 1.032) | 0.055 | |  |
|  | Heart24 | 1 | Reference |  | |  |
|  |  | 2 | 0.901 (0.547 ~ 1.484) | 0.682 | |  |
|  |  | 3 | 2.001 (1.183 ~ 3.385) | 0.010 | |  |
|  |  | 4 | 1.727 (0.762 ~ 3.914) | 0.190 | |  |
|  | Heart48 | 1 | Reference |  | |  |
|  |  | 2 | 1.188 (0.735 ~ 1.921) | 0.482 | |  |
|  |  | 3 | 1.784 (1.058 ~ 3.009) | 0.030 | |  |
|  |  | 4 | 2.042 (0.944 ~ 4.415) | 0.070 | |  |
|  | Heart72 | 1 | Reference |  | |  |
|  |  | 2 | 1.722 (0.980 ~ 3.026) | 0.059 | |  |
|  |  | 3 | 2.205 (1.208 ~ 4.025) | 0.010 | |  |
|  |  | 4 | 3.708 (1.812 ~ 7.588) | <0.001 | |  |
| **βBlock** |  |  |  |  | |  |
| No (n=283) | 24h average |  | 1.049 (1.014 ~ 1.085) | 0.006 | | 0.058 |
|  | Heart24 | 1 | Reference |  | | 0.045 |
|  |  | 2 | 1.783 (0.686 ~ 4.632) | 0.235 | |  |
|  |  | 3 | 5.198 (1.531 ~ 17.651) | 0.008 | |  |
|  |  | 4 | 4.352 (0.913 ~ 20.731) | 0.065 | |  |
|  | Heart48 | 1 | Reference |  | | 0.082 |
|  |  | 2 | 2.139 (0.819 ~ 5.584) | 0.120 | |  |
|  |  | 3 | 3.122 (0.937 ~ 10.402) | 0.064 | |  |
|  |  | 4 | 5.566 (1.274 ~ 24.317) | 0.022 | |  |
|  | Heart72 | 1 | Reference |  | | 0.203 |
|  |  | 2 | 2.449 (0.858 ~ 6.990) | 0.094 | |  |
|  |  | 3 | 2.028 (0.578 ~ 7.119) | 0.270 | |  |
|  |  | 4 | 10.620 (2.847 ~ 39.615) | <0.001 | |  |
| Yes (n=974) | 24h average |  | 1.012 (0.996 ~ 1.028) | 0.145 | |  |
|  | Heart24 | 1 | Reference |  | |  |
|  |  | 2 | 0.618 (0.394 ~ 0.969) | 0.036 | |  |
|  |  | 3 | 1.092 (0.679 ~ 1.754) | 0.717 | |  |
|  |  | 4 | 1.468 (0.715 ~ 3.016) | 0.296 | |  |
|  | Heart48 | 1 | Reference |  | |  |
|  |  | 2 | 0.830 (0.536 ~ 1.286) | 0.404 | |  |
|  |  | 3 | 1.055 (0.652 ~ 1.706) | 0.828 | |  |
|  |  | 4 | 1.635 (0.821 ~ 3.256) | 0.162 | |  |
|  | Heart72 | 1 | Reference |  | |  |
|  |  | 2 | 1.180 (0.704 ~ 1.978) | 0.531 | |  |
|  |  | 3 | 1.268 (0.726 ~ 2.214) | 0.404 | |  |
|  |  | 4 | 2.394 (1.233 ~ 4.651) | 0.010 | |  |
| **Inpatient days** |  |  |  |  | |  |
| <=7  (n=770) | 24h average |  | 1.003 (0.984 ~ 1.021) | 0.775 | | 0.149 |
|  | Heart24 | 1 | Reference |  | | 0.146 |
|  |  | 2 | 1.002 (0.584 ~ 1.718) | 0.994 | |  |
|  |  | 3 | 1.635 (0.903 ~ 2.960) | 0.105 | |  |
|  |  | 4 | 1.517 (0.517 ~ 4.451) | 0.448 | |  |
|  | Heart48 | 1 | Reference |  | | 0.129 |
|  |  | 2 | 1.203 (0.723 ~ 2.001) | 0.476 | |  |
|  |  | 3 | 1.204 (0.661 ~ 2.194) | 0.544 | |  |
|  |  | 4 | 1.623 (0.587 ~ 4.486) | 0.351 | |  |
|  | Heart72 | 1 | Reference |  | | 0.635 |
|  |  | 2 | 1.633 (0.931 ~ 2.863) | 0.087 | |  |
|  |  | 3 | 1.516 (0.803 ~ 2.863) | 0.199 | |  |
|  |  | 4 | 3.322 (1.442 ~ 7.651) | 0.005 | |  |
| >7  (n=487) | 24h average |  | 1.036 (1.016 ~ 1.057) | <0.001 | |  |
|  | Heart24 | 1 | Reference |  | |  |
|  |  | 2 | 0.543 (0.289 ~ 1.020) | 0.058 | |  |
|  |  | 3 | 1.032 (0.538 ~ 1.980) | 0.925 | |  |
|  |  | 4 | 2.000 (0.844 ~ 4.739) | 0.115 | |  |
|  | Heart48 | 1 | Reference |  | |  |
|  |  | 2 | 0.755 (0.394 ~ 1.448) | 0.398 | |  |
|  |  | 3 | 1.251 (0.636 ~ 2.462) | 0.516 | |  |
|  |  | 4 | 2.873 (1.222 ~ 6.753) | 0.015 | |  |
|  | Heart72 | 1 | Reference |  | |  |
|  |  | 2 | 1.086 (0.487 ~ 2.420) | 0.840 | |  |
|  |  | 3 | 1.428 (0.613 ~ 3.325) | 0.409 | |  |
|  |  | 4 | 3.110 (1.250 ~ 7.736) | 0.015 | |  |

Adjusting for variables such as age, gender, BMI, SBP, DBP, Smoking, Drinking, Hypertension, COPD, Atrial Fibrillation, Tumor, Myocardiopathy, Diabetes, Stroke, Old Myocardial Infarction, Killip class, NT-proBNP, LDL, TG, Creatinine, Expired Myocardial Infarction, LVEF, LM, LAD, LCX, RA, Other Branches, Inpatient Days, cTNT, CK-MB,βBlock, CCB, and ACEI/ARB

A3. Delong Test

|  | **Z** | ***P*-value** |
| --- | --- | --- |
| 24h average **vs.** heart 24 | -0.358 | 0.720 |
| 24h average **vs.** heart 48 | 0.099 | 0.920 |
| 24h average **vs.** heart 72 | -0.855 | 0.392 |
| heart 24 **vs.** heart 48 | 0.617 | 0.537 |
| heart 24 **vs.** heart 72 | -0.343 | 0.731 |
| heart 48 **vs.** heart 72 | -1.025 | 0.305 |

Table S8. Delong Test
